# Long-Term Survival and Cancer Risk in the Hepatitis C Virus-Infected Patients After Antiviral Treatment: A Nationwide Cohort Study

**DOI:** 10.7150/jca.87259

**Published:** 2024-01-01

**Authors:** Yi-Ju Chen, Jing-Yang Huang, Rathinasamy Baskaran, Mosleh Mohammad Abomughaid, Chang-Chi Hsieh, Wan-Teng Lin

**Affiliations:** 1 Department of Surgery, Taichung Veterans General Hospital, Taichung 40704, Taiwan.; 2 Department of Animal Science and Biotechnology, Tunghai University, Taichung 40704, Taiwan.; 3 Center for health data science, Chung Shan Medical University Hospital, Taichung, Taiwan.; 4 Institute of Medicine, College of Medicine, Chung Shan Medical University, Taichung, Taiwan.; 5 Department of Bioinformatics and Medical Engineering, Asia University, Taichung 413305, Taiwan.; 6 Department of Medical Laboratory Sciences, College of Applied Medical Sciences, University of Bisha, Bisha 61922, Saudi Arabia.; 7 Department of Hospitality Management, College of Agriculture, Tunghai University, Taichung 407224, Taiwan.; 8 R&D Division, Utopia Holiday Hotel Corporation, Taichung, Taiwan.

## Abstract

**Background:** Exposure to the Hepatitis C virus (HCV) has been identified as one of the most critical risk factors for Hepatocellular carcinoma (HCC). Interferons and direct-acting antivirals (DAAs) have been used to treat HCV infection with high rates (95%) of prolonged virological response, a suitable safety profile, and good compliance rates.

**Methods:** We obtained information from Taiwan's Health and Welfare Data Science Center. (HWDSC). In this observational cohort research, patients with HCV who received a diagnosis in Taiwan between 2011 and 2018 were included.

**Results:** 78,300 untreated HCV patients were paired for age, sex, and index date with 39,150 HCV patients who received interferon or DAAs treatment. Compared to the control group, the Interferon or DAAs treatment sample has fewer low-income individuals and more hospitalization requirements. The percentage of kidney illness was reduced in the therapy group compared to the control group, but the treatment group had a greater comorbidity rate of gastric ulcers. Interferon or DAA therapy for HCV-infected patients can substantially lower mortality. All cancer diagnoses after HCV infection with interferon treatment aHR 95% CI = 0.809 (0.774-0.846), Sofosbuvir-based DAA aHR 95% CI = 1.009 (0.737-1.381) and Sofosbuvir free DAA aHR 95% CI = 0.944 (0.584-1.526) showing cancer-protective effects in the INF-treated cohort but not DAA.

**Conclusion:** Following antiviral therapy, women appear to have a more substantial preventive impact than men against pancreatic, colorectal, and lung cancer. Interferon or DAAs treatment effect was more significant in the cirrhotic group.

## Introduction

Hepatocellular carcinoma (HCC) is a common form of malignancy across the world. The occurrence of HCC has been steadily increasing both worldwide and in Taiwan 1. Chronic hepatitis C virus (HCV) infection is the leading cause of mortality related to liver disease and the primary indication for liver transplantation. Recent estimates indicate that the number of individuals worldwide suffering from HCV is around 71.1 million, with approximately 1.75 million cases arising annually 2. About 400,000 fatalities are attributed to chronic HCV infections annually, primarily due to hepatic diseases 3. HCV is a single-stranded RNA virus with 9.6 kb nucleotide belonging to the family *Flaviviridae*. HCV predominantly infects hepatocytes due to the expression of key entry receptors and liver-specific cellular host components necessary for viral replication 4, 5. Induced intracellular oxidative stress damage caused by viral proteins; deregulation of cell signalling pathways by viral proteins such as HBx, L-HDAg, S-HDAg, HCV core, NS3, and NS5A/B); and persistent liver inflammation and immune-mediated oxidative stress damage from chronic viral infection are all critical factors of the HCV induced HCC 4.

HCC is regularly observed in livers exhibiting histological abnormalities, presenting an opportunity for the development and growth of tumors in the presence of chronic hepatic disease. It has been indicated that approximately 90% of instances of HCC possess a correlative risk factor 6. The progress of HCC is primarily attributed to the presence of cirrhosis in the liver, regardless of the cause. This is widely regarded as a pre-cancerous lesion and has been observed in over 70% of HCC cases 7. After all extended liver ailments, cirrhosis of the liver is observed, with chronic Hepatitis B Virus (HBV) or HCV infection being the most pervasive causative agent 8. Results from previous studies suggest that HCV may be associated with certain extrahepatic solid tumors, including cancers of the stomach, gastrointestinal tract, colorectal, pancreas, prostate, breast, kidney, and lung; however, the evidence is inadequate and conflicting across the studies conducted 9-11.

Antiviral treatment has been shown to lower the likelihood of such liver disease development when chronic HCV infection is successfully eliminated. Interferon-alpha (IFN)-based regimens were the mainstay of previous antiviral therapies. But pegylated IFN (PegIFN)/ribavirin (RBV) has many side effects and needs to be taken for a long time (at least 24 to 48 weeks), so a high number of people have to stop treatment. As a result, its widespread usage is somewhat constrained, especially for patients with concomitant conditions like decompensated cirrhosis or advanced age 12. Their sustained virological response (SVR) rates are also below average, which is more significant. The development of PegIFN-free direct-acting antivirals (DAAs) in 2014 has made it possible to achieve an SVR rate > 95% with an acceptable tolerability 13.

The advent of the currently approved IFN-free regimens, which comprise all-oral direct-acting antivirals (DAAs) targeting viral proteins such as the NS3/4A protease, NS5B polymerase and the NS5A replication complex, have revolutionized the management of HCV infection given their high efficacy based on SVR rates of over 95%, as well as their good safety profiles and exceptional tolerability 13, 14. The expectation was that viral clearance would result in a lower morbidity and mortality rate, thus reducing the risk of developing hepatocellular carcinoma. The majority of HCV infections can be remedied through the utilization of DAA agents. DAAs are better tolerated and more efficacious than traditional interferon (IFN)-based therapies, with more than 95% of treated patients achieving HCV clearance 15. It is salient to note that patients with HCV who are treated with DAAs have a significantly decreased danger of progressing to the HCC 13. It remains unclear if interferon or DAA therapy reduces the risk of extrahepatic malignancies; however, it is plausible. Analysis of specific research has suggested that the introduction of DAAs in individuals suffering from HCV infection has decreased extrahepatic (outside of the liver) conditions, such as complications of the cardiovascular, metabolic, renal, and haematological systems. This indicates that the benefits of treating HCV with DAAs are not limited to the liver 16, 17. The findings are corroborated by research into the effects of IFN-based treatments beyond the liver, which suggests that those who respond well to treatment have a lower likelihood of lymphoid neoplasms and gastrointestinal malignancies in comparison to those who remain untreated in terms of HCV 10, 18.

Despite the potential for the emergence of hepatic and extrahepatic cancers after an HCV infection, there is a shortage of research examining the ramifications of interferon or DAAs therapy following an HCV infection and the likelihood of other cancers developing within the population. Consequently, the dynamics of HCV infection and associated treatments' consequences are poorly understood. The available data regarding carcinogenesis in HCV-infected individuals is scant. To the extent of our knowledge, no studies have surfaced that compare the incidence of cancer in individuals receiving interferon and interferon-free DAA therapy after the initial curative treatment of HCV. As far as we know, it is the first study to utilize a nationwide cohort study in the Taiwanese population.

## Materials and Methods

### Data source

Our retrospective cohort study involved 23 million participants enrolled in Taiwan's Health Insurance Program from 2010 to 2018. We obtained data from the Health and Welfare Data Science Center, which maintains various nationwide databases in Taiwan for academic research purposes. To assess the risk of cancer or mortality in patients with chronic HCV infection after treatment with Interferon or DAAs, we linked three nationwide databases: the National Health Insurance Research Database (NHIRD), Cancer Registry Database, and Death Registry Records 19. We used registry data and medical claims of inpatient and outpatient visits to identify personal demographics, potential disease diagnosis, prescription, and surgery from the NHIRD. The Cancer Registry Database has existed since 1979 and provides high-quality data on validated registry frameworks, including data on cancer diagnosis. The Death Registry Records allowed us to determine survival status, date of death, and cause of death. All participants were assigned a unique and hashed personal identification number to link data between the nationwide databases. The study was approved by the Institutional Review Board of Chung Shan Medical University Hospital's Human Research Ethics Committee (CS1-20201).

### Definition of the study population

**Figure 1:**
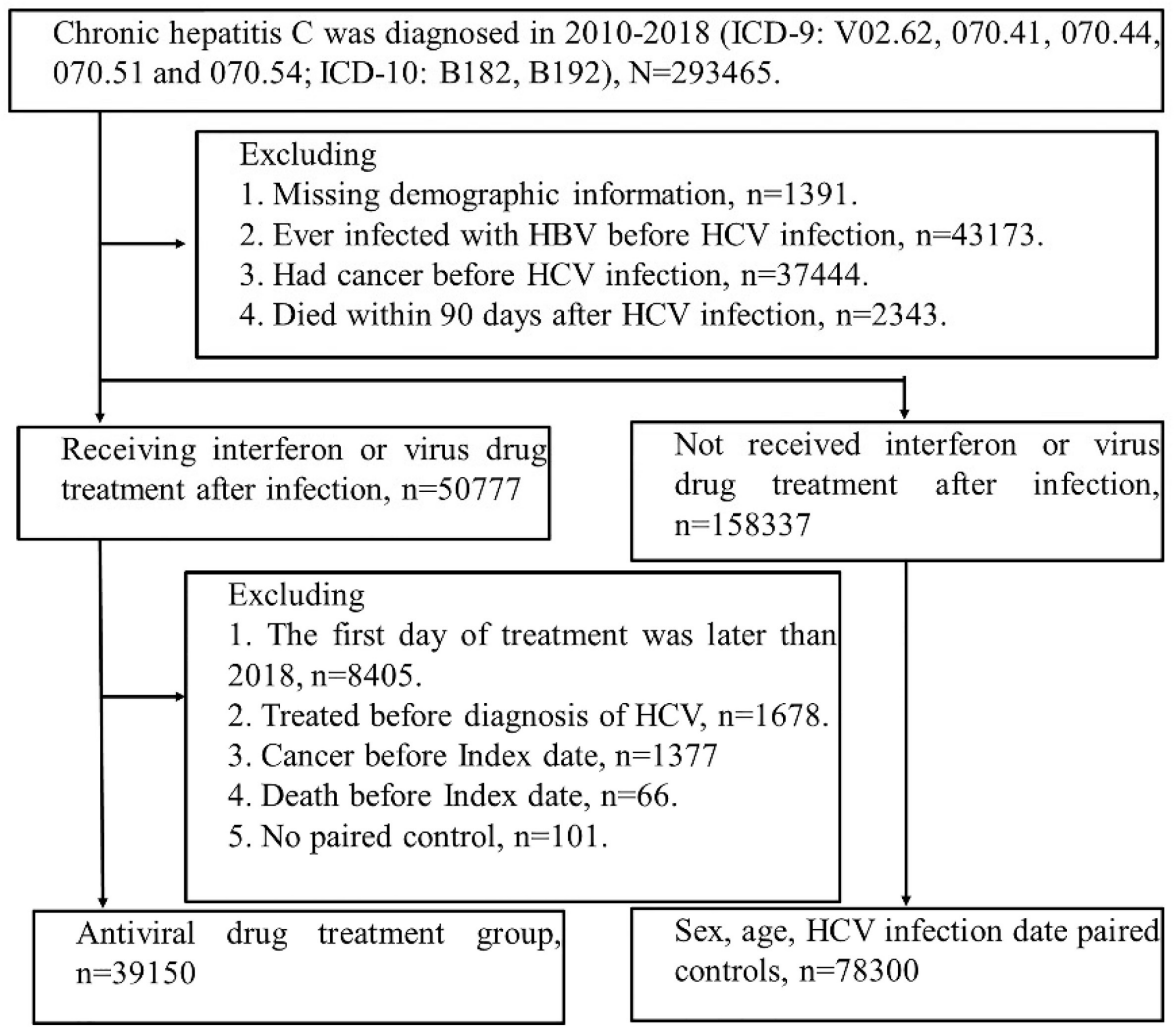
** Study population flow chart.** The figure shows people with HCV infection who were included in the study. Index date of treatment group = first treatment day + 90 days. 1. Interferon (INF) has health insurance benefits (2010-2018). 2. Sofosbuvir-based direct-acting antivirals (DAAs), (Health insurance benefits only available after 2017). 3. Sofosbuvir-free DAAs, (health insurance benefits will be available after 2017). Finally, according to age (±2 year), sex, and hepatitis diagnosis date (within 6 months), the patients were paired (Index = treatment + 90 days). 78,300 people with suspected hepatitis C infection were selected without viral drug treatment, and 39,150 people were treated with viral drug.

We identified the patients newly diagnosed with HCV infection (ICD-9 codes: V02.62, 070.41, 070.44, 070.51 or 070.54; ICD-10: B17.1, B18.2) between 2010 and 2018. Initially, there were 293465 patients selected, and the patients who had missing demographics data, co-infected with HBV, cancer, or dead within three months after HCV infection were excluded. Among 209114 patients, 50777 patients received Interferon or DAAs after HCV infection. We defined the index date as the three months after treatment of Interferon or DAAs to avoid potential surveillance or detection bias. Furthermore, we excluded the patients who had treatment for HCV, dead or had any diagnosis of cancer before the index date. There were 39150 patients involved in the interferon or DAAs cohort, and 78300 comparisons were matched by age, sex and index date. **(Figure 1)**.

### Definition of study covariates

We obtained baseline demographics such as age, sex, urbanization, and insured category from the registry file. Age was calculated in years by subtracting the birth date from the index date, and patients were categorized into seven age groups. To identify comorbidities, we used ICD-9-CM codes listed in Supplementary Table 1, which were recorded within one year before the index date. Comorbidities included Hypertension, Diabetes mellitus, Hyperlipidemia, Renal disease, Osteoporosis, Osteoarthritis, Ischemic heart disease, Stroke, COPD, Dementia, Peptic ulcer, Liver cirrhosis, Inflammatory bowel diseases, Gastrointestinal bleeding, Choledocholithiasis, Cholangitis, or Helicobacter infection.

### Identification of study event

The subsequent mortality and cancer risks were identified from the information on the Death Registry or Cancer Registry Database. The patients who were newly diagnosed with cancer, including Gastric cancer, liver (including biliary) cancer, liver cancer, biliary cancer, pancreatic cancer, colorectal cancer, lung cancer, oral cancer, breast cancer (female), prostate cancer (male), were ascertained by using the ICD-9 codes **(Supplementary Table 1)**.

### Statistical Analysis

In this large-sample observational study, we employed the absolute standardized difference (ASD) in order to compare the baseline Scovariates between groups 20. The qualities exhibited equilibrium when the Autocorrelation Spatial Distance was less than 0.1. We utilized survival models to analyze the correlation between mortality and cancer risk with HCV treatment. The Poisson distribution was used to determine the incidence rate and 95% confidence interval (CI). For the duration of the study, all participants were tracked from the initial date to the point of the occurrence of the study event. The cutoff point encompassed mortality or completion of the investigation (31DEC2018). Kaplan-Meier plots were utilized to evaluate the 7-year cumulative cancer incidence between the treatment and non-treatment groups. A log-rank test was employed to assess the homogeneity of the hazard rate functions across the various study groups. The proportional hazard assumption was tested, and univariate and multivariable Cox proportional hazards regression analyses were performed to assess the hazard ratio (HR) of exposure to HCV treatment on cancer risk.

In the multivariable regression, we took into account the covariates, which consisted of the index year and baseline demographics (sex, age, urbanization, insured category) as well as comorbidities (Hypertension, Diabetes mellitus, Hyperlipidemia, Renal disease, Osteoporosis, Osteoarthritis, Ischemic heart disease, Stroke, COPD, Dementia, Peptic ulcer, Liver cirrhosis, Inflammatory bowel diseases, Gastrointestinal bleeding, Choledocholithiasis, Cholangitis, or Helicobacter infection). The HR was estimated utilizing the subdistribution Fine-Gray regression approach, taking mortality as the competing event. Utilizing multivariate Cox regression, a subgroup assessment and an interaction effect test were conducted to assess the impact of sex and age across different stratifications. Statistical analyses were carried out utilizing SAS version 9.4. (SAS Institute, Cary, NC, USA). For the hypothesis test, a significance level of 0.05 was employed.

## Results

### Patients Characteristics

Table 1 shows the baseline characteristics of the 2 study groups. The covariates have been well-balanced between the 2 study groups after propensity score matching. After applying the exclusion criteria, we documented 78300 interferon or DAA-treated patients and 39150 without treatment among the 117,450 individuals diagnosed with HCV from 2011 to 2018. Most patients who started Interferon or DAA therapy are in late middle age (50-59). The length of hospital stay (> six days) the patients spent in the hospital was significantly higher in the Interferon or DAA-treated groups compared to the control group. Interferon or DAAs treatment cohort paired by age, gender, and Index date has a lower proportion of low-income and more hospitalization needs than the control generation. The treatment group had a higher comorbidity rate of gastric ulcers, but the proportion of kidney disease was lesser than that of the control group.

### Cancer incidence among HCV-infected people with and without interferon or DAA treatment

**Figure 2:**
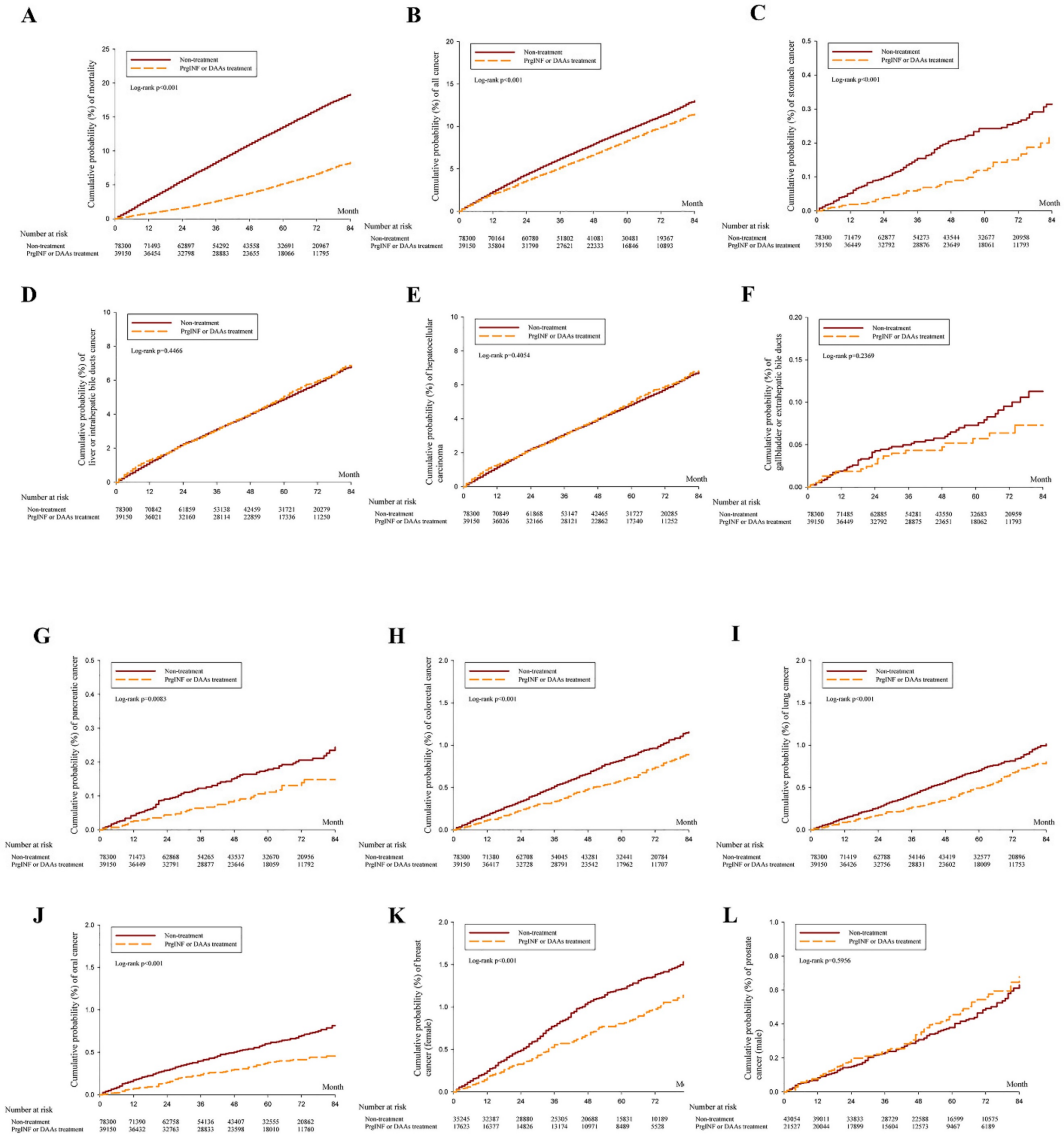
7-year cumulative cancer incidence between the treatment and non-treatment groups was plotted using the KM curve. (A) All cause mortality, (B) All cancer mortality, (C) Stomach cancer, (D) Liver (biliary) cancer, (E) Hepatocellular carcinoma, (F) Galbladder cancer, (G) Pancreatic cancer, (H) Colorectal cancer, (I) Lung cancer, (J) Oral cancer, (K) Breast cancer, (L) Prostate cancer. aHR (95% CI): Adjusted hazard ratios with 95% confidence intervals.

The study included 3,980,041 person-month of follow-ups for people without interferon or DAA treatment and 2,103,987 person-month of follow-up for people receiving interferon or DAA treatment for HCV. Table 2 shows that patients with HCV infection receiving Interferon or DAAs treatment can significantly reduce mortality aHR=0.414 (95% CI = 0.394-0.435), All cancer aHR = 0.813 (0.778-0.850), gastric cancer aHR = 0.541 (0.387-0.757), liver cancer aHR = 0.919 (0.867-0.975), pancreatic cancer aHR = 0.618 (0.429-0.890), colorectal cancer aHR = 0.722 (0.616-0.847), lung cancer aHR = 0.740 (0.624-0.879), oral cancer aHR = 0.573 (0.467-0.702), female breast cancer aHR = 0.679 (0.555-0.829). 7-year cumulative cancer incidence between the treatment and non-treatment groups was plotted using the KM curve (Figure 2).

### Subgroup analysis

**Figure 3:**
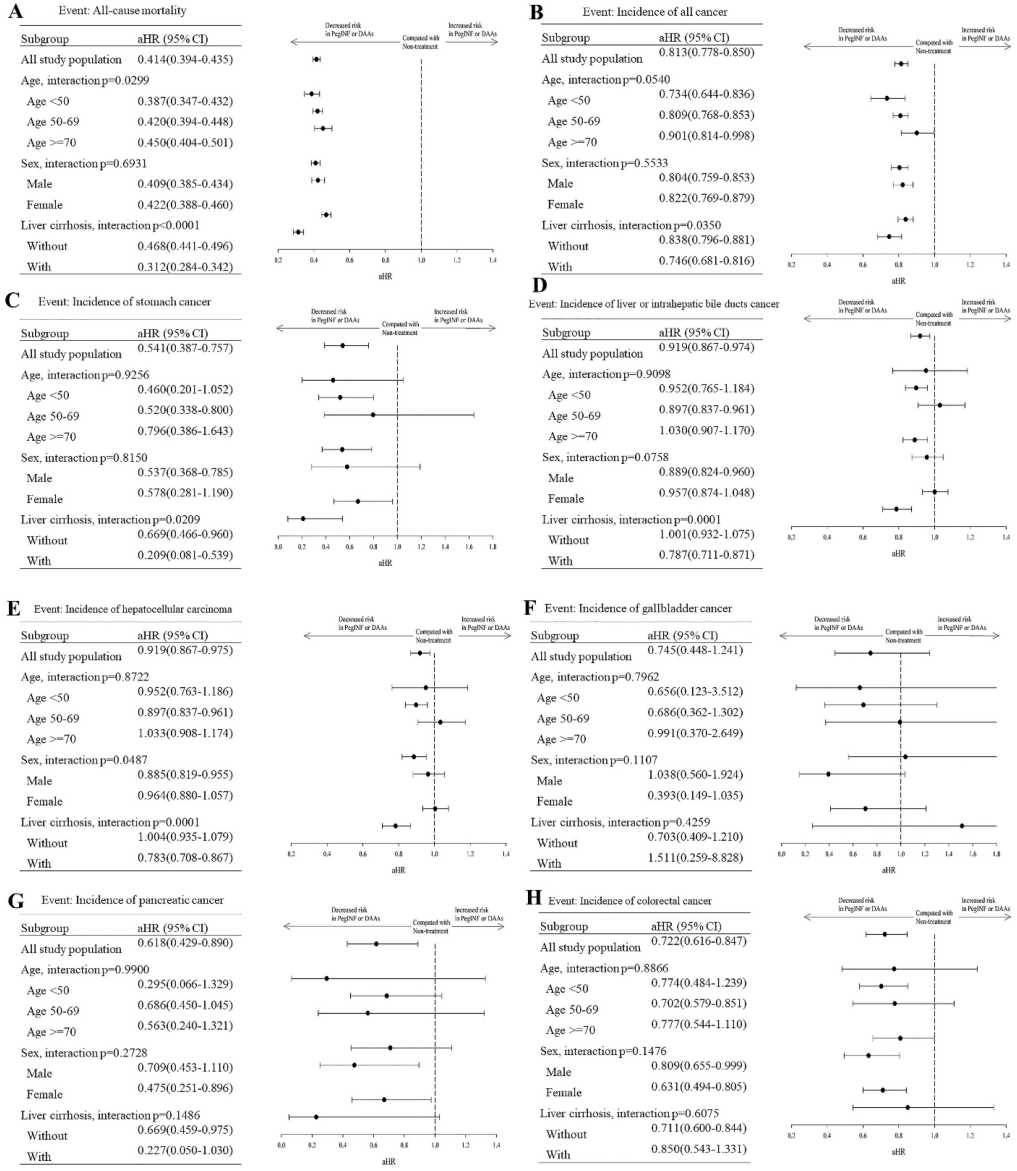

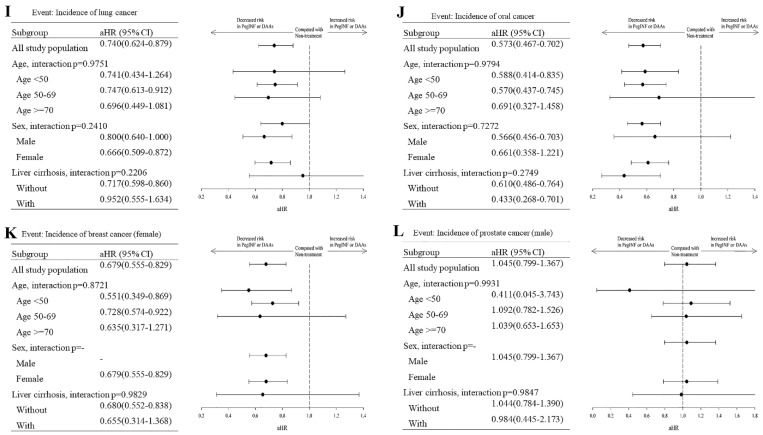
Subgroup distribution analysis of mortality and cancer incidence between the treatment and non-treatment groups. (A) All cause mortality, (B) All cancer mortality, (C) Stomach cancer, (D) Liver (biliary) cancer, (E) Hepatocellular carcinoma, (F) Galbladder cancer, (G) Pancreatic cancer, (H) Colorectal cancer, (I) Lung cancer, (J) Oral cancer, (K) Breast cancer, (L) Prostate cancer. aHR (95% CI): Adjusted hazard ratios with 95% confidence intervals.

Subgroup analysis of cancer development in HCV patients with and without interferon or DAA treatment was done based on age, sex, and cirrhosis (Figure 3). The age stratification results showed that younger people (< 50) receiving Interferon or DAAs treatment had a better effect. But the impact of liver cancer in the 50-69 age is better. There were no significant differences in gender stratification. Women seem to have a better protective effect against pancreatic, colorectal, and lung cancer than males after antiviral treatment. Interferon or DAAs treatment effect was more significant in the cirrhotic group.

### Effect of different antiviral drugs (interferon, Sofosbuvir based DAA and Sofosbuvir free DAA) on HCV patients

Since DAA-based treatment was introduced in 2017, interferons have been used for HCV treatment. Further detailed analysis was done by comparing different drugs used for the HCV infection (interferon, Sofosbuvir based DAA and Sofosbuvir free DAA). Table 3 shows that most individuals received INF because DAA treatment only started to use from 2017, so there is a time difference. The mortality risk event without treatment was 9646, for interferon was 1910 (aHR (95% CI)-0.413), Sofosbuvir based DAA was 17 (aHR (95% CI)- 0.330), and Sofosbuvir free DAA was 15 (aHR (95% CI)- 0.605) (Table 4). Whereas all cancer diagnoses after HCV infection with interferon treatment aHR 95% CI = 0.809 (0.774-0.846), Sofosbuvir-based DAA aHR 95% CI = 1.009 (0.737-1.381) and Sofosbuvir free DAA aHR 95% CI = 0.944 (0.584-1.526) showing cancer-protective effects in the INF-treated cohort but not DAA.

## Discussion

This retrospective cohort study aimed to analyze the effects of IFN and DAA therapies on overall survival rates and incidence of all cancers among HCV-infected individuals. In addition to causing liver disease, HCV is linked to a wide variety of other illnesses, all contributing to an increased risk of death. Among the various extrahepatic manifestations associated with HCV, mixed cryoglobulinemia (MC), non-Hodgkin's lymphomas (NHL), heart disease, renal failure, insulin resistance, type 2 diabetes mellitus, neurological and psychiatric disorders, and non-MC rheumatic diseases are the most widely reported 21. The impacts on these persons' morbidity, quality of life, and mortality may be influenced by the manifestation of extrahepatic symptoms 22.

In patients without severe virological responses or who were left untreated, interferon-based treatment significantly slowed the development of the illness and its complications, including HCC, in pre-cirrhotic and cirrhotic patients who achieved severe virological response 23, 24. Older age, male gender, advanced liver cirrhosis, fatty liver, and a high posttreatment serum alpha-fetoprotein (AFP) level are risk factors for HCC formation in IFN-treated individuals who achieve SVR 25, 26. However, IFN-based therapy was not the best option for treating individuals with chronic HCV infection because of the restrictive inclusion criteria, poor severe virological response rates, and significant therapy-associated toxicity 13.

The inhibitory effects of interferon-based therapy on hepatocarcinogenesis in the context of HCV infection have already been demonstrated. Additionally, several randomized controlled studies 27-29 and meta-analyses 30, 31 have shown that interferon-based therapy increased the overall survival rate in patients getting curative treatment for HCV-associated HCC and avoided HCC recurrence. It has been observed that the overall mortality rate of individuals receiving PEG-IFN is higher compared to those who did not receive interferon treatment, and the post-curative recurrence rate of hepatocellular carcinoma amongst patients achieving substantial virologic responses was significantly lower in comparison to those who did not receive interferon. Notably, survival rates were constant irrespective of whether or not PEG-IFN patients in the sustained virological response group or PEG-IFN patients without the SVR cohort had disparate rates of first and second occurrences of liver cancer 32. A comparable outcome was found for the PEG-IFN + ribavirin combination treatment 33.

The introduction of interferon-free regimens comprised of novel DAAs has undoubtedly been a significant development in managing patients with persisting HCV infections, showcasing robust virological response rates in combination with an acceptable level of tolerance. This has raised expectations for averting consequences like liver cancer and other severe liver problems in HCV patients. These hypotheses are supported by data from previous research done using the interferon for HCV treatment, which showed a decrease in liver cancer frequency in patients who achieved a severe virological response 34, 35.

Kanwal et al. performed a cohort analysis that assessed the danger of HCC in a population of 22500 individuals undergoing DAA therapy and followed them for an average of 1.02 years. A study revealed a remarkable decrease in the danger of HCC in those with SVR in correlation to those without it, with a rate of 0.90 and 3.45 HCC/100 person-year (PY), respectively; after accounting for adjustment, the HR was 0.28, with a 95% confidence interval of 0.22-0.36 36. Calleja et al. conducted a study analyzing almost 4000 HCV patients undergoing DAAs treatment at various locations around Spain; the research sought to determine the efficacy, safety and clinical outcomes associated with DAA-based therapy in those infected with HCV genotype 1. The results of the study revealed an incidence of 0.93% of hepatocellular carcinoma (HCC) within 18 months of beginning treatment with the combination of ombitasvir/paritaprevir/ritonavir plus dasabuvir and ledipasvir/sofosbuvir 37. A previous cohort study reported a decreased risk of liver cancer in individuals with chronic HCV infection who had been treated with DAAs 36. Results of another retrospective study reported long-term risk of HCC in patients who had achieved SVR to DAAs and were evaluated for 3.5 years after SVR 38. Among eighteen-thousand-seventy-six patients who achieved sustained virologic response with direct-acting antivirals, it was revealed that five-hundred-forty-four individuals developed *de novo* hepatocellular carcinoma, manifesting cumulative risks of 1.1%, 1.9%, and 2.8% after one, two, and three years, respectively. The findings from two recently released studies concord with those reported in the Kanwal et al. 38 study. In a retrospective analysis, Tani et al. found that the total rates of hepatocellular carcinoma over the preceding 12 and 36 months were 1.88 and 6.00%, respectively 39. Another study by Watanabe et al. revealed that the 1- and 2-year accumulated instances of liver cancer were 1.9% and 4.1%, respectively 40.

A trial of U.S. troops discovered that while interferon-mediated HCV cure was associated with significant drops in hematologic malignancies, similar results were not seen in patients treated with DAA to elicit HCV cure 18. This implies that the data on cancer risk in patients receiving interferon-based treatment might not be comparable to those receiving DAAs. A deeper understanding of how DAA therapy may impact the risk of extrahepatic cancers may be essential for HCV clinical care and cancer monitoring after HCV cure as more people with the disease will be healed within the next ten years 41. There is evidence that the clinical profile of HCV-infected individuals is changing during the DAA era, as seen in the ecologic data of HCV-related mortality rates, which have significantly decreased between pre- and post-DAA ages 42, 43. However, mortality rates from extrahepatic cancers during the DAA era have increased, signifying that, as opposed to HCC-related deaths, which DAAs have effectively curbed, death due to extrahepatic cancers has risen 44.

An analysis using propensity score matching found no significant difference between interferon-based and interferon-free treatment groups (5-year incidence: 54.2% in interferon-based, 45.1% in interferon-free therapy; P = 0.54) in a retrospective cohort study comparing outcomes of patients with prior HCV related liver cancer treated with DAAs vs. interferon-based therapy 45. The results of a recent retrospective cohort conducted in Japan were consistent with the initial findings, showing that therapy with interferon had a similar effect in decreasing the risk of liver cancer recurrence compared to therapy with interferon-based treatments (P = 0.564) 46.

An increased risk of liver cancer and other extrahepatic cancers, such as non-Hodgkin's lymphoma has been linked to HCV infection. HCV infection has been linked to the development of some extrahepatic solid tumors, such as lung, pancreatic, oral/oropharyngeal, and anal cancers, thus raising the issue of the potential necessity for heightened cancer surveillance in those who are cured of HCV infection. Treatment with interferon has been demonstrated to be correlated with a reduction in the risk of certain liver and hematologic cancers.

## Supplementary Material

Supplementary table.

## Figures and Tables

**Table 1:** Baseline characteristics among study groups.

	Comparison	Interferon or DAAs treatment	ASD
N	78300	39150	
**Index year**			0.0000
2011-2014	49082 (62.68%)	24541 (62.68%)	
2015-2018	29218 (37.32%)	14609 (37.32%)	
**Sex**			0.0000
Male	43054 (54.99%)	21527 (54.99%)	
Female	35246 (45.01%)	17623 (45.01%)	
**Age**			0.0000
<30	1678 (2.14%)	871 (2.22%)	
30-39	8556 (10.93%)	4300 (10.98%)	
40-49	15253 (19.48%)	7612 (19.44%)	
50-59	24054 (30.72%)	12065 (30.82%)	
60-69	20526 (26.21%)	10299 (26.31%)	
70-79	7332 (9.36%)	3567 (9.11%)	
>=80	901 (1.15%)	436 (1.11%)	
**Urbanization**			0.0446
Urban	39402 (50.32%)	19119 (48.84%)	
Sub-urban	26218 (33.48%)	13591 (34.72%)	
Rural	12680 (16.19%)	6440 (16.45%)	
**Insured category**			0.1815
Government	2958 (3.78%)	1281 (3.27%)	
Privately held company	38835 (49.60%)	20372 (52.04%)	
Agricultural organizations	17009 (21.72%)	8940 (22.84%)	
Low-income	1353 (1.73%)	402 (1.03%)	
Non-labor force	17222 (21.99%)	7709 (19.69%)	
Others	923 (1.18%)	446 (1.14%)	
**Length of hospital stay, days**			0.1585
0	58476 (74.68%)	31279 (79.90%)	
1-6	8791 (11.23%)	4485 (11.46%)	
>6	11033 (14.09%)	3386 (8.65%)	
**Co-morbidity**			
Hypertension	27997 (35.76%)	13749 (35.12%)	0.0133
Diabetes mellitus	17718 (22.63%)	8689 (22.19%)	0.0104
Hyperlipidemia	17512 (22.37%)	7874 (20.11%)	0.0551
Renal disease	6605 (8.44%)	2025 (5.17%)	0.1299
Osteoporosis	2356 (3.01%)	1005 (2.57%)	0.0268
Osteoarthritis	12891 (16.46%)	6035 (15.42%)	0.0286
Ischemic heart disease	7594 (9.70%)	3522 (9.00%)	0.0241
Stroke	4041 (5.16%)	1399 (3.57%)	0.0777
COPD	5272 (6.73%)	2484 (6.34%)	0.0157
Dementia	892 (1.14%)	287 (0.73%)	0.0422
Peptic ulcer	17341 (22.15%)	10351 (26.44%)	0.1002
Liver cirrhosis	6858 (8.76%)	4185 (10.69%)	0.0652
Inflammatory bowel diseases	660 (0.84%)	304 (0.78%)	0.0074
Gastrointestinal bleeding	4938 (6.31%)	2266 (5.79%)	0.0218
Choledocholithiasis	3418 (4.37%)	2164 (5.53%)	0.0536
Cholangitis	479 (0.61%)	199 (0.51%)	0.0139
Helicobacter infection	690 (0.88%)	511 (1.31%)	0.0408

ASD, absolute standardized difference, when ASD >0.1, means that the variable has a difference between the two groups. Table 1 shows that the Interferon or DAAs treatment cohort paired by age, gender and Index date has a lower proportion of low-income and more hospitalization needs than the control generation. The treatment group had a higher comorbidity rate of gastric ulcer, but the proportion of kidney disease was lower than that of the control group.

**Table 2:** Risk of study events in study groups.

	Comparison	Interferon or DAAs treatment
N	78300	39150
**Mortality risk**		
Person-month	3980041	2103987
event	9646	1942
Mortality rate (per 1000 person-months)	24.24 (23.76-24.72)	9.23 (8.83-9.65)
Crude Hazard Ratio (95% CI)	Reference	0.380(0.362-0.399)
Adjusted Hazard Ratio (95% CI)	Reference	0.414(0.394-0.435)
**All cancers**		
Person-month	3820959	2020407
event	6481	2949
Rate (per 1000 person-months)	16.96 (16.55-17.38)	14.60 (14.08-15.13)
Crude Hazard Ratio (95% CI)	Reference	0.863 (0.826-0.901)
Adjusted Hazard Ratio (95% CI)	Reference	0.813 (0.778-0.850)
Competing HR (95% CI)	Reference	0.865(0.828-0.904)
**Stomach cancer**		
Person-month	3977327	2103103
event	155	45
Rate (per 1000 person-months)	0.39(0.33-0.46)	0.21(0.16-0.29)
Crude Hazard Ratio (95% CI)	Reference	0.550(0.395-0.766)
Adjusted Hazard Ratio (95% CI)	Reference	0.541 (0.387-0.757)
Competing HR (95% CI)	Reference	0.587 (0.419-0.822)
**Liver (biliary) cancer**		
Person-month	3907178	2052661
event	3321	1780
Rate (per 1000 person-months)	8.50(8.22-8.79)	8.67(8.28-9.08)
Crude Hazard Ratio (95% CI)	Reference	1.023 (0.965-1.083)
Adjusted Hazard Ratio (95% CI)	Reference	0.919 (0.867-0.974)
Competing HR (95% CI)	Reference	0.998(0.940-1.058)
**Liver cancer**		
Person-month	3907778	2053020
event	3280	1762
Rate (per 1000 person-months)	8.39 (8.11-8.69)	8.58(8.19-8.99)
Crude Hazard Ratio (95% CI)	Reference	1.025 (0.967-1.086)
Adjusted Hazard Ratio (95% CI)	Reference	0.919 (0.867-0.975)
Competing HR (95% CI)	Reference	0.998(0.941-1.060)
**Gallbladder cancer**		
Person-month	3979268	2103598
event	54	Twenty-one
Rate (per 1000 person-months)	0.14 (0.10-0.18)	0.10(0.07-0.15)
Crude Hazard Ratio (95% CI)	Reference	0.739 (0.446-1.223)
Adjusted Hazard Ratio (95% CI)	Reference	0.745(0.448-1.241)
Competing HR (95% CI)	Reference	0.786(0.472-1.309)
**Pancreatic cancer**		
Person-month	3978582	2103532
event	120	39
Rate (per 1000 person-months)	0.30(0.25-0.36)	0.19(0.14-0.25)
Crude Hazard Ratio (95% CI)	Reference	0.618 (0.431-0.887)
Adjusted Hazard Ratio (95% CI)	Reference	0.618 (0.429-0.890)
Competing HR (95% CI)	Reference	0.649 (0.447-0.944)
**Colorectal cancer**		
Person-month	3963480	2097278
event	553	215
Rate (per 1000 person-months)	1.40(1.28-1.52)	1.03(0.90-1.17)
Crude Hazard Ratio (95% CI)	Reference	0.736(0.629-0.862)
Adjusted Hazard Ratio (95% CI)	Reference	0.722 (0.616-0.847)
Competing HR (95% CI)	Reference	0.761 (0.650-0.891)
**Lung cancer**		
Person-month	3971446	2100449
event	469	186
Rate (per 1000 person-months)	1.18 (1.08-1.29)	0.89(0.77-1.02)
Crude Hazard Ratio (95% CI)	Reference	0.748(0.631-0.886)
Adjusted Hazard Ratio (95% CI)	Reference	0.740(0.624-0.879)
Competing HR (95% CI)	Reference	0.779 (0.656-0.926)
**Oral Cancer**		
Person-month	3969875	2100528
event	405	123
Rate (per 1000 person-months)	1.02(0.93-1.12)	0.59(0.49-0.70)
Crude Hazard Ratio (95% CI)	Reference	0.578 (0.473-0.707)
Adjusted Hazard Ratio (95% CI)	Reference	0.573 (0.467-0.702)
Competing HR (95% CI)	Reference	0.573 (0.467-0.702)
**Breast cancer (Female)**	N=35246	N=17623
Person-month	1849709	960939
event	371	132
Rate (per 1000 person-months)	2.01(1.81-2.22)	1.37(1.16-1.63)
Crude Hazard Ratio (95% CI)	Reference	0.689 (0.564-0.840)
Adjusted Hazard Ratio (95% CI)	Reference	0.679 (0.555-0.829)
Competing HR (95% CI)	Reference	0.697(0.570-0.853)
**Prostate Cancer (Male)**	N=43054	N=21527
Person-month	2113380	1136226
event	148	86
Rate (per 1000 person-months)	0.70(0.60-0.82)	0.76(0.61-0.93)
Crude Hazard Ratio (95% CI)	Reference	1.075 (0.824-1.402)
Adjusted Hazard Ratio (95% CI)	Reference	1.045 (0.799-1.367)
Competing HR (95% CI)	Reference	1.120(0.856-1.466)

Table 2 shows that patients with HCV infection receiving Interferon or DAAs treatment can significantly reduce Mortality aHR=0.414 (95% CI=0.394-0.435), All cancer aHR=0.813 (0.778-0.850), gastric cancer aHR=0.541 (0.387-0.757), liver cancer aHR=0.919 (0.867-0.975), pancreatic cancer aHR=0.618 (0.429-0.890), colorectal cancer aHR=0.722 (0.616-0.847), lung cancer aHR=0.740 (0.624-0.879), oral cancer aHR=0.573 (0.467-0.702), female breast cancer aHR=0.679 (0.555-0.829).

**Table 3:** Basic Information on the HCV-infected patients treated without treatment with interferon, Sofosbuvir based DAA and Sofosbuvir free DAA.

	No treatment	INF	Sofosbuvir based DAA	Sofosbuvir free DAA
N	78300	36928	1258	964
**Index year**				
2011-2014	49082 (62.68%)	24541 (66.46%)	0 (0.00%)	0 (0.00%)
2015-2018	29218 (37.32%)	12387 (33.54%)	1258 (100.00%)	964 (100.00%)
**Sex**				
Male	43054 (54.99%)	20550 (55.65%)	515 (40.94%)	462 (47.93%)
Female	35246 (45.01%)	16378 (44.35%)	743 (59.06%)	502 (52.07%)
**Age**				
<30	1678 (2.14%)	868 (2.35%)	3 (0.24%)	0 (0.00%)
30-39	8556 (10.93%)	4262 (11.54%)	17 (1.35%)	21 (2.18%)
40-49	15253 (19.48%)	7405 (20.05%)	110 (8.74%)	97 (10.06%)
50-59	24054 (30.72%)	11611 (31.44%)	232 (18.44%)	222 (23.03%)
60-69	20526 (26.21%)	9589 (25.97%)	451 (35.85%)	259 (26.87%)
70-79	7332 (9.36%)	2998 (8.12%)	320 (25.44%)	249 (25.83%)
>=80	901 (1.15%)	195 (0.53%)	125 (9.94%)	116 (12.03%)
**Urbanization**				
Urban	39402 (50.32%)	18118 (49.06%)	568 (45.15%)	433 (44.92%)
Sub-urban	26218 (33.48%)	12819 (34.71%)	435 (34.58%)	337 (34.96%)
Rural	12680 (16.19%)	5991 (16.22%)	255 (20.27%)	194 (20.12%)
**Attributes of the insured unit**				
Public security	2958 (3.78%)	1226 (3.32%)	27 (2.15%)	28 (2.90%)
Labor protection	38835 (49.60%)	19339 (52.37%)	599 (47.62%)	434 (45.02%)
Farmers' Association, Water Conservancy, Fisherman's Association	17009 (21.72%)	8251 (22.34%)	405 (32.19%)	284 (29.46%)
Low-income households	1353 (1.73%)	377 (1.02%)	10 (0.79%)	15 (1.56%)
Public office for insurance	17222 (21.99%)	7315 (19.81%)	203 (16.14%)	191 (19.81%)
Other	923 (1.18%)	420 (1.14%)	14 (1.11%)	12 (1.24%)
**The number of days in hospital**				
0	58476 (74.68%)	29614 (80.19%)	957 (76.07%)	708 (73.44%)
1-6	8791 (11.23%)	4198 (11.37%)	160 (12.72%)	127 (13.17%)
>6	11033 (14.09%)	3116 (8.44%)	141 (11.21%)	129 (13.38%)
**Co-morbidity**				
Hypertension	27997 (35.76%)	12620 (34.17%)	646 (51.35%)	483 (50.10%)
Diabetes mellitus	17718 (22.63%)	8003 (21.67%)	398 (31.64%)	288 (29.88%)
Hyperlipidemia	17512 (22.37%)	7419 (20.09%)	283 (22.50%)	172 (17.84%)
Renal disease	6605 (8.44%)	1669 (4.52%)	202 (16.06%)	154 (15.98%)
Osteoporosis	2356 (3.01%)	867 (2.35%)	73 (5.80%)	65 (6.74%)
Osteoarthritis	12891 (16.46%)	5555 (15.04%)	264 (20.99%)	216 (22.41%)
Ischemic heart disease	7594 (9.70%)	3243 (8.78%)	160 (12.72%)	119 (12.34%)
Stroke	4041 (5.16%)	1320 (3.57%)	34 (2.70%)	45 (4.67%)
COPD	5272 (6.73%)	2307 (6.25%)	94 (7.47%)	83 (8.61%)
Dementia	892 (1.14%)	187 (0.51%)	52 (4.13%)	48 (4.98%)
PUDs	17341 (22.15%)	9738 (26.37%)	353 (28.06%)	260 (26.97%)
Liver cirrhosis	6858 (8.76%)	3574 (9.68%)	350 (27.82%)	261 (27.07%)
Inflammatory bowel diseases	660 (0.84%)	298 (0.81%)	3 (0.24%)	3 (0.31%)
Gastrointestinal bleeding	4938 (6.31%)	1940 (5.25%)	174 (13.83%)	152 (15.77%)
Choledocholithiasis	3418 (4.37%)	2024 (5.48%)	87 (6.92%)	53 (5.50%)
Cholangitis	479 (0.61%)	179 (0.48%)	13 (1.03%)	7 (0.73%)
Helicobacter infection	690 (0.88%)	483 (1.31%)	12 (0.95%)	16 (1.66%)

It can be observed from Table 4 that most of them still belong to INF treatment, because DAA has only started to pay since 2017, so there is a time difference.

**Table 4:** Time to the event of the HCV-infected patients treated without treatment with interferon, Sofosbuvir based DAA, and Sofosbuvir free DAA.

	No treatment	INF	Sofosbuvir based DAA	Sofosbuvir free DAA
N	78300	36928	1258	964
**Mortality risk**				
Person-month	3980041	2088519	10997	4471
event	9646	1910	17	15
cHR (95%CI)	Reference	0.377(0.359-0.396)	0.661 (0.410-1.065)	1.393 (0.838-2.317)
aHR (95% CI)	Reference	0.413 (0.393-0.434)	0.330(0.205-0.533)	0.605(0.363-1.008)
**All cancers**				
Person-month	3820959	2005249	10732	4426
event	6481	2892	40	17
cHR (95%CI)	Reference	0.854 (0.817-0.892)	1.918 (1.404-2.622)	1.864 (1.156-3.007)
aHR (95% CI)	Reference	0.809 (0.774-0.846)	1.009 (0.737-1.381)	0.944 (0.584-1.526)
Competing HR (95% CI)	Reference	0.863 (0.825-0.902)	1.016 (0.740-1.396)	0.906(0.559-1.467)
**Liver (biliary) cancer**				
Person-month	3907178	2037420	10799	4442
event	3321	1741	27	12
cHR (95%CI)	Reference	1.009(0.952-1.069)	2.488 (1.700-3.643)	2.481 (1.403-4.388)
aHR (95% CI)	Reference	0.914 (0.862-0.970)	1.057 (0.721-1.551)	1.000(0.564-1.772)
Competing HR (95% CI)	Reference	0.996(0.939-1.057)	1.078 (0.731-1.588)	0.944 (0.530-1.679)
**Liver cancer**				
Person-month	3907778	2037773	10805	4442
event	3280	1725	25	12
cHR (95%CI)	Reference	1.012 (0.955-1.073)	2.335(1.572-3.469)	2.518 (1.424-4.452)
aHR (95% CI)	Reference	0.916 (0.863-0.971)	0.984(0.661-1.465)	1.007(0.568-1.786)
Competing HR (95% CI)	Reference	0.998(0.940-1.060)	1.004 (0.672-1.501)	0.951 (0.534-1.692)

cHR (95%CI), crude hazard ratios with 95% confidence intervals; aHR (95% CI), adjusted hazard ratios with 95% confidence intervals. Cancer-protective effects was observed in the INF-treated cohort but not DAA.

## References

[1] Chen DS (2007). Hepatocellular carcinoma in Taiwan. Hepatology Research.

[2] Hollande C, Parlati L, Pol S (2020). Micro-elimination of hepatitis C virus. Liver International.

[3] Moon AM, Singal AG, Tapper EB (2020). Contemporary epidemiology of chronic liver disease and cirrhosis. Clinical Gastroenterology and Hepatology.

[4] D'souza S, Lau KC, Coffin CS, Patel TR (2020). Molecular mechanisms of viral hepatitis induced hepatocellular carcinoma. World journal of gastroenterology.

[5] Zhao P, Malik S, Xing S (2021). Epigenetic mechanisms involved in HCV-induced hepatocellular carcinoma (HCC). Frontiers in Oncology.

[6] Ohata K, Hamasaki K, Toriyama K, Matsumoto K, Saeki A, Yanagi K (2003). Hepatic steatosis is a risk factor for hepatocellular carcinoma in patients with chronic hepatitis C virus infection. Cancer.

[7] Santhakumar C, Gane EJ, Liu K, McCaughan GW (2020). Current perspectives on the tumor microenvironment in hepatocellular carcinoma. Hepatology International.

[8] Alberts CJ, Clifford GM, Georges D, Negro F, Lesi OA, Hutin YJ (2022). Worldwide prevalence of hepatitis B virus and hepatitis C virus among patients with cirrhosis at country, region, and global levels: a systematic review. The Lancet Gastroenterology & Hepatology.

[9] Hwang JP, LoConte NK, Rice JP, Foxhall LE, Sturgis EM, Merrill JK (2019). Oncologic implications of chronic hepatitis C virus infection. Journal of oncology practice.

[10] Masarone M, Persico M (2019). Hepatitis C virus infection and non-hepatocellular malignancies in the DAA era: A systematic review and meta-analysis. Liver International.

[11] Hong SW, Choi W-M, Hwang HW, Kim DS, Yoon J, Lee JW (2021). Chronic viral hepatitis is associated with colorectal neoplasia: A systematic review and meta-analysis. Digestive Diseases and Sciences.

[12] Tahata Y, Sakamori R, Urabe A, Yamada R, Ohkawa K, Hiramatsu N (2020). Clinical outcomes of direct-acting antiviral treatments for patients with hepatitis C after hepatocellular carcinoma are equivalent to interferon treatment. Hepatology Research.

[13] Muzica CM, Stanciu C, Huiban L, Singeap A-M, Sfarti C, Zenovia S (2020). Hepatocellular carcinoma after direct-acting antiviral hepatitis C virus therapy: A debate near the end. World journal of gastroenterology.

[14] Mohanty A, Salameh S, Butt AA (2019). Impact of direct acting antiviral agent therapy upon extrahepatic manifestations of hepatitis C virus infection. Current HIV/AIDS Reports.

[15] Bourlière M, Bronowicki J-P, De Ledinghen V, Hézode C, Zoulim F, Mathurin P (2015). Ledipasvir-sofosbuvir with or without ribavirin to treat patients with HCV genotype 1 infection and cirrhosis non-responsive to previous protease-inhibitor therapy: a randomised, double-blind, phase 2 trial (SIRIUS). The Lancet Infectious Diseases.

[16] Cabibbo G, Celsa C, Calvaruso V, Petta S, Cacciola I, Cannavò MR (2019). Direct-acting antivirals after successful treatment of early hepatocellular carcinoma improve survival in HCV-cirrhotic patients. Journal of hepatology.

[17] Reig M, Mariño Z, Perelló C, Iñarrairaegui M, Ribeiro A, Lens S (2016). Unexpected high rate of early tumor recurrence in patients with HCV-related HCC undergoing interferon-free therapy. Journal of hepatology.

[18] Ioannou GN, Green PK, Berry K, Graf SA (2019). Eradication of hepatitis C virus is associated with reduction in hematologic malignancies: major differences between interferon and direct-acting antivirals. Hepatology Communications.

[19] Hsieh C-Y, Su C-C, Shao S-C, Sung S-F, Lin S-J, Kao Yang Y-H (2019). Taiwan's national health insurance research database: past and future. Clinical epidemiology.

[20] Austin PC (2009). Balance diagnostics for comparing the distribution of baseline covariates between treatment groups in propensity-score matched samples. Statistics in medicine.

[21] Mazzaro C, Quartuccio L, Adinolfi LE, Roccatello D, Pozzato G, Nevola R (2021). A review on extrahepatic manifestations of chronic hepatitis C virus infection and the impact of direct-acting antiviral therapy. Viruses.

[22] Negro F, Forton D, Craxì A, Sulkowski MS, Feld JJ, Manns MP (2015). Extrahepatic morbidity and mortality of chronic hepatitis C. Gastroenterology.

[23] Morgan RL, Baack B, Smith BD, Yartel A, Pitasi M, Falck-Ytter Y (2013). Eradication of hepatitis C virus infection and the development of hepatocellular carcinoma: a meta-analysis of observational studies. Annals of internal medicine.

[24] Bruno S, Di Marco V, Iavarone M, Roffi L, Crosignani A, Calvaruso V (2016). Survival of patients with HCV cirrhosis and sustained virologic response is similar to the general population. Journal of Hepatology.

[25] Asahina Y, Tsuchiya K, Nishimura T, Muraoka M, Suzuki Y, Tamaki N (2013). α-fetoprotein levels after interferon therapy and risk of hepatocarcinogenesis in chronic hepatitis C. Hepatology.

[26] El-Serag HB, Kanwal F, Richardson P, Kramer J (2016). Risk of hepatocellular carcinoma after sustained virological response in Veterans with hepatitis C virus infection. Hepatology.

[27] Lo CM, Liu CL, Chan SC, Lam CM, Poon RT, Ng IO (2007). A randomized, controlled trial of postoperative adjuvant interferon therapy after resection of hepatocellular carcinoma. Annals of surgery.

[28] Mazzaferro V, Romito R, Schiavo M, Mariani L, Camerini T, Bhoori S (2006). Prevention of hepatocellular carcinoma recurrence with alpha-interferon after liver resection in HCV cirrhosis. Hepatology.

[29] Lin SM, Lin CJ, Hsu CW, Tai DI, Sheen IS, Lin DY (2004). Prospective randomized controlled study of interferon-alpha in preventing hepatocellular carcinoma recurrence after medical ablation therapy for primary tumors. Cancer.

[30] Singal A, Freeman Jr D, Anand B (2010). Meta-analysis: interferon improves outcomes following ablation or resection of hepatocellular carcinoma. Alimentary pharmacology & therapeutics.

[31] Miyake Y, Iwasaki Y, Yamamoto K (2010). Meta-analysis: reduced incidence of hepatocellular carcinoma in patients not responding to interferon therapy of chronic hepatitis C. International journal of cancer.

[32] Hagihara H, Nouso K, Kobayashi Y, Iwasaki Y, Nakamura S, Kuwaki K (2011). Effect of pegylated interferon therapy on intrahepatic recurrence after curative treatment of hepatitis C virus-related hepatocellular carcinoma. International journal of clinical oncology.

[33] Kawaoka T, Hayes CN, Ohishi W, Ochi H, Maekawa T, Abe H (2011). Predictive value of the IL28B polymorphism on the effect of interferon therapy in chronic hepatitis C patients with genotypes 2a and 2b. Journal of hepatology.

[34] van der Meer AJ, Veldt BJ, Feld JJ, Wedemeyer H, Dufour J-F, Lammert F (2012). Association between sustained virological response and all-cause mortality among patients with chronic hepatitis C and advanced hepatic fibrosis. Jama.

[35] D'Ambrosio R, Della Corte C, Colombo M (2015). Hepatocellular carcinoma in patients with a sustained response to anti-hepatitis C therapy. International journal of molecular sciences.

[36] Kanwal F, Kramer J, Asch SM, Chayanupatkul M, Cao Y, El-Serag HB (2017). Risk of hepatocellular cancer in HCV patients treated with direct-acting antiviral agents. Gastroenterology.

[37] Calleja JL, Crespo J, Rincón D, Ruiz-Antorán B, Fernandez I, Perelló C (2017). Effectiveness, safety and clinical outcomes of direct-acting antiviral therapy in HCV genotype 1 infection: Results from a Spanish real-world cohort. Journal of hepatology.

[38] Kanwal F, Kramer JR, Asch SM, Cao Y, Li L, El-Serag HB (2020). Long-term risk of hepatocellular carcinoma in HCV patients treated with direct acting antiviral agents. Hepatology.

[39] Tani J, Morishita A, Sakamoto T, Takuma K, Nakahara M, Fujita K (2020). Simple scoring system for prediction of hepatocellular carcinoma occurrence after hepatitis C virus eradication by direct-acting antiviral treatment: All Kagawa Liver Disease Group Study. Oncology Letters.

[40] Watanabe T, Tokumoto Y, Joko K, Michitaka K, Horiike N, Tanaka Y (2020). Sex difference in the development of hepatocellular carcinoma after direct-acting antiviral therapy in patients with HCV infection. Journal of Medical Virology.

[41] Chhatwal J, Wang X, Ayer T, Kabiri M, Chung RT, Hur C (2016). Hepatitis C disease burden in the United States in the era of oral direct-acting antivirals. Hepatology.

[42] Kim D, Li AA, Gadiparthi C, Khan MA, Cholankeril G, Glenn JS (2018). Changing trends in etiology-based annual mortality from chronic liver disease, from 2007 through 2016. Gastroenterology.

[43] Kim D, Li AA, Perumpail BJ, Gadiparthi C, Kim W, Cholankeril G (2019). Changing trends in etiology-based and ethnicity-based annual mortality rates of cirrhosis and hepatocellular carcinoma in the United States. Hepatology.

[44] Kim D, Adejumo AC, Yoo ER, Iqbal U, Li AA, Pham EA (2019). Trends in mortality from extrahepatic complications in patients with chronic liver disease, from 2007 through 2017. Gastroenterology.

[45] Nagata H, Nakagawa M, Asahina Y, Sato A, Asano Y, Tsunoda T (2017). Effect of interferon-based and-free therapy on early occurrence and recurrence of hepatocellular carcinoma in chronic hepatitis C. Journal of hepatology.

[46] Imai K, Takai K, Hanai T, Suetsugu A, Shiraki M, Shimizu M (2020). Sustained virological response by direct-acting antivirals reduces the recurrence risk of hepatitis C-related hepatocellular carcinoma after curative treatment. Molecular and clinical oncology.

