# Supplementary material for: Long-Term Survival and Cancer Risk in the Hepatitis C Virus-Infected Patients After Antiviral Treatment: A Nationwide Cohort Study
Source: J Cancer. 2024 Jan 1;15(1):113–25. doi: 10.7150/jca.87259 (PMC10751673; doi:10.7150/jca.87259)
Supplement: Supplementary file 1 — Supplementary table. [file jcav15p0113s1.pdf]

Table S1: Definition of co-morbidities and study outcome.

Variable	Definition by ICD-9 codes	Definition by ICD-10 codes
**Co-morbidity (**Records in NHIRD)		
Hypertension	401-405	I10-I15
Diabetes mellitus	250	E08-E13
Hyperlipidemia	272	E71, E75, E77, E78, E88
Renal diseases	582-586	N03, N05-N07, N16-N19, E10.21, E11.21
Osteoporosis	733.0	M81, M80, M84.4-M84.6
Osteoarthritis	715	M15-M19
Ischemic heart disease	410-414	I20-I25
Stroke	430-438	I60-I63
COPD	491, 492, 496	J41-J44
Dementia	290, 294.1, 331.0	F01-F05, G30, G31, G91
Peptic ulcer	531, 532, 533	K25, K31.82, K56.60, K26, K27
Liver cirrhosis	571.2 571.5 571.6	K70.2 K74
Inflammatory bowel diseases	555, 556	K50, K51
Gastrointestinal bleeding	578	K25.0, K26.0, K27.0, K28.0, K29.0
Choledocholithiasis	574	K80
Cholangitis	576.1, 576.2	K83.0, K83.1
Helicobacter infection	041.86	B96.81
**Outcome (**Records in cancer registry database or NHIRD)		

All cancer	140-208	C00-C97
Gastric cancer	151	C16
Liver cancer including intrahepatic bile ducts	155	C22
Liver cancer	155.0, 155.2	C22.x except for C22.1
Gallbladder cancer	156	C23
Pancreatic cancer	157	C25
Colorectal cancer	153, 154	C18-C20
Lung cancer	162	C33, C34
Lip, oral cavity, and pharynx cancer	140-149	C00-C14
Female breast cancer	174	C50 in female
Prostate cancer	185	C61
